# Usability Evaluation of the Preoperative ISBAR (Identification, Situation, Background, Assessment, and Recommendation) Desktop Virtual Reality Application: Qualitative Observational Study

**DOI:** 10.2196/40400

**Published:** 2022-12-29

**Authors:** Eva Mari Andreasen, Rune Høigaard, Helen Berg, Aslak Steinsbekk, Kristin Haraldstad

**Affiliations:** 1 Department of Health and Nursing Sciences University of Agder Kristiansand Norway; 2 Department of Sport Science and Physical Education University of Agder Kristiansand Norway; 3 Department of Health Sciences Norwegian University of Science and Technology Ålesund Norway; 4 Department of Public Health and Nursing Norwegian University of Science and Technology Trondheim Norway

**Keywords:** desktop virtual reality, handover, ISBAR, preoperative, undergraduate nursing students, usability evaluation, usability, nursing, health care education, student, medical education, medical training, VR, virtual reality, surgery, surgical, System Usability Scale, communication, self-instruction, self-guided, nurse, training, undergraduate, health care professional, health care provider

## Abstract

**Background:**

Systematic communication, such as the ISBAR (identification, situation, background, assessment, recommendation) approach, comprises a generic, transferable nontechnical skill. It can be used during the handover of patients set to undergo surgery and can be practiced in various ways, including virtual reality (VR). VR increasingly has been implemented and valued in nursing education as a positive contribution to teach students about pre- and postoperative nursing. A new nonimmersive 3D learning activity called the Preoperative ISBAR Desktop VR Application has been developed for undergraduate nursing students to learn preoperative handover using the ISBAR approach. However, the usability of this learning activity has not been studied.

**Objective:**

This study aimed to investigate how second-year undergraduate nursing students evaluated the usability of the Preoperative ISBAR Desktop VR Application.

**Methods:**

This was a qualitative study with observation and interviews. The inclusion criteria were undergraduate second-year nursing students of varying ages, gender, and anticipated technological competence. The System Usability Scale (SUS) questionnaire was used to get a score on overall usability.

**Results:**

A total of 9 second-year nursing students aged 22-29 years participated in the study. The average score on the SUS was 83 (range 0-100), which equals a “B” on the graded scale and is excellent for an adjective-grade rating. The students expressed increased motivation to learn while working in self-instructed desktop VR. Still, a few technical difficulties occurred, and some students reported that they experienced some problems comprehending the instructions provided in the application. Long written instructions and a lack of self-pacing built into the application were considered limitations.

**Conclusions:**

The nursing students found the application to be usable overall, giving it an excellent usability score and noting that the application provided opportunities for active participation, which was motivational and facilitated their perceived learning outcomes. The next version of the application, to be used in a randomized controlled trial, will be upgraded to address technological and comprehension issues.

## Introduction

The exchange of relevant clinical information from one provider to another (eg, handover) is crucial for the surgical pathway because missing information and incomplete handover can lead to adverse patient outcomes [1,2]. The ISBAR (identification, situation, background, assessment, and recommendation) approach is an evidence-based approach to ensure consistent, structured communication [3] and can be used in inter- and intraprofessional collaboration for patients about to undergo surgery [4-8]. Studies have reported that using ISBAR can improve communication between health care providers [9,10] and reduce communication errors [11].

Considering that a lack of clear communication directly or indirectly can endanger patient safety, the evidence suggests that ISBAR skills acquisition should start early in nursing education [12,13]. ISBAR traditionally is learned through role-playing in simulations or in classroom settings [14,15]. The past few years have seen an increased interest in virtual reality (VR) as a method to learn structured communication [16-18].

Desktop VR is a computer-generated 3D environment presented on nonimmersive desktop and laptop PC screens [19]. Desktop VR typically is built around user interaction, such as moving avatars, typing commands, and interacting with others while completing a task [19]. The advantage of desktop VR is that it has potential for letting users practice without supervision while receiving audio and visual instruction and has instant feedback from the VR application itself in a safe environment [17]. VR has been increasingly implemented and valued in nursing education as a positive contribution to curricula to teach students about pre- and postoperative nursing [20-22]. Using desktop VR as an active learning method also aligns with studies that have recommended interactive teaching strategies in curricula [23]. However, to the best of our knowledge, no published research exists on desktop VR solutions that practice handover using the ISBAR approach in a preoperative setting [24].

Perceived usability is essential when developing such solutions [25-27]. The International Organization for Standardization has defined *usability* as “the extent to which a product can be used by specified users to achieve specified goals with effectiveness, efficiency, and satisfaction in a specified context of use” [28]. Furthermore, the degree of learnability is defined as part of the usability assessment [28]. We developed the Preoperative ISBAR Desktop VR Application (henceforth “application”), which is intended to be used in a randomized controlled trial. Thus, its usability needs to be tested to optimize the application for virtual simulation in nursing education.

This study aimed to investigate how second-year undergraduate nursing students evaluated the application’s usability.

## Methods

### Design

This was a qualitative observational study with interviews. The usability test was conducted during the fall semester of 2021.

### Preoperative ISBAR Desktop VR application

The application was part of a research project focusing on the use of VR in health care education [29]. It was created to teach handover skills when using the ISBAR approach and is based on cognitive principles from the 4-component instructional design (4C/ID) [30] guidelines, comprising (1) a learning task, (2) supportive information, (3) procedural information, and (4) part-task practice. Instructions and tasks were based on evidence-based knowledge of learner-centered teaching [31-33] and national ISBAR guidelines [34]. A version of the application still under development was used.

The various sequences in the application are presented in Table 1. The students were organized into groups of 3 who played together in VR through 3 main activities. The first activity was to sort patient information individually using the ISBAR approach and compare and discuss the participants' individual sorting to clarify the ISBAR approach. The second activity was to perform handovers using the ISBAR approach, which was between a nurse on a night shift and a day shift, and between a nurse on a day shift and an anesthesia nurse. The third activity was a debriefing that focused on the experience in general and on selecting the most important patient information to communicate first.

**Table 1:** Presentation of the Preoperative ISBAR^a^ Desktop VR Application.

Number	Sequence	Content
1	Instruction: register name and select group number	A screen with a visible square to insert the participant’s name. Group allocation number visible with instruction to choose groups
2	Instruction: introduction to ISBAR	Animation with a voiceover explaining briefly what ISBAR is, presenting the learning objectives, and providing a brief overview of the tasks
3	Task: familiarization with desktop VR^b^ and each other	A screen displays instructions on how to use the arrow keys to look around in the desktop VR and introduce the players to each other
4	Instruction: sort patient information	Animation with a voiceover instructing how to sort single pieces of patient information according to the ISBAR approach and how to get additional information in pop-up windows
5	Task: sort patient information	A screen with an area displaying 1 piece of patient information, buttons for each ISBAR letter to select where the patient information should be sorted, and a list containing patient information sorted in the order of the selected ISBAR letters with the opportunity to delete the patient information and to sort it again. An explanation of the ISBAR approach is available as a pop-up
6	Task: discussion of experience with sorting	A screen displays a comparison of how each participant sorted the patient information and a suggestion for correct sorting. The percentage of patient information sorted similarly to the suggested solution is displayed for each player
7	Instruction and task: patient case and choose a role	Animation with a voiceover presenting a patient case, the 3 roles involved (nurse on night shift, nurse on day shift, and nurse anesthetist), and how to choose a role
8	Instruction and task: role description and choose a role	A screen with a written description of the 3 roles involved and pictures symbolizing the roles to be clicked to select a role. When a player clicks on a role, the frame changes to green for that player and red for the other players
9	Instruction: handover role play	Animation with a voiceover instructing how to complete the next task and a handover role play in which participants give and receive patient information in their active roles (nurse on night shift, nurse on day shift, and nurse anesthetist) using the ISBAR approach. Instruction on active participation for both the giver and receiver in handover (sender starts with the selected patient information, and the receiver requests additional patient information)
10	Instruction: handover role play	A screen displays a written summary of the next task
11	Task: handover role play	A screen displays a list of all patient information and a virtual phone. The text states that the player should select the patient information to be presented first and then call the next nurse to perform the handover through the virtual phone. The phone and handover checklist are visible to the receiver of the handover. An explanation of the role play is available as a pop-up for all players during handover practice. The participant's screen with the active role is visible to the other participants in the group. ISBAR explanation available as a pop-up
12	Instruction: debriefing 1	Short animation with a voiceover describing what should be done during the debriefing session
13	Task: debriefing 1	Text stating that they should discuss each participant’s experience doing the tasks in general and that they will discuss each participant in detail afterward
14	Instruction: debriefing 2	Animation with a voiceover with instructions to debrief what each participant chose to highlight and say first during the handover
15	Task: debriefing 2	A screen displays a list of all patient information, with the patient information that the participant had clicked on as the information to present first in bold (highlighted). Suggested bullet points on what to discuss during the debriefing are visible as a pop-up explanation. An ISBAR explanation is available as a pop-up
16	Instruction: debriefing closure	Animation with a voiceover with encouragement to practice again
17	Task: final practice and ending	A screen with available options: to practice again or end the practice. If selecting to practice again, it starts at sequence 2

^a^ISBAR: identification, situation, background, assessment, and recommendation.

^b^VR: virtual reality.

### Participants and Recruitment

The aim was to include undergraduate second-year nursing students with variations in age, gender, and anticipated technological competence. With 3 students participating in each group, 9 participants were viewed as adequate for robust usability to get a measure of the perceived usability and to get a good assessment of how people see a system or a product [35].

Information about the study was presented verbally in a compulsory lecture for the second-year nursing students at a university in Norway. Furthermore, written study information and recruitment invitations were displayed on a web-based notice board. Those interested were asked to contact the study, and if they did, they received more detailed information about the study, and an appointment for a test time was set. The students were assigned to the 3 groups based on the order in which they signed up for the study.

### Procedure and Data Collection

#### Overview

The whole learning activity comprised watching a 9-minute video introducing ISBAR [36] and practicing within the application. Three students in each group were placed in separate rooms to ensure that all communication happened in the application, mimicking a situation in which the students were in different locations. One researcher was present in each room to observe and provide support if needed.

Data were collected through (1) background questions, (2) observation, (3) the System Usability Scale (SUS), and (4) focus group interviews.

#### Background Questions

The participants were asked about their gender, age, and whether they had participated in compulsory ISBAR teaching (yes/no). The participants were also asked about their self-reported technological competence, measured on a 4-point graded scale developed for this study, ranging from level 1 (*low competence*) to level 4 (*high competence*).

#### Observation

The students were encouraged to think aloud, that is, verbalize their thoughts, constantly [37] while using the application. The think-aloud sessions were video-recorded, and field notes were taken based on a predefined observation template covering navigation errors, ease of use, apparent misunderstandings, and technical difficulties (Multimedia Appendix 1). If the students were unsure of how to proceed with the application, they were encouraged to do what they would find most intuitive before being assisted, as Rubin and Chisnell [25] recommended.

#### System Usability Scale

The participants were asked to complete the SUS [38] after they finished using the application. SUS is a recommended tool for evaluating educational technology systems [26], comprising 10 open-ended items with 5 answer options ranging from 1 (*strongly disagree*) to 5 (*strongly agree*). The final mean score ranges from 0 to 100, and the score can be reported as an A-F grade using a curved grading scale [39], and as an adjective score, ranging from *worst imaginable* to *best imaginable* [40].

#### Focus Group Interviews

After completing the SUS, a focus group interview was conducted with each group. An interview guide—which was developed based on the research question, predefined observation template, and usability theory [25]—was used (Multimedia Appendix 2). Examples of questions asked included, “What did you like the most about learning Preoperative ISBAR in desktop VR?,” “What did you like the least about learning Preoperative ISBAR in desktop VR?,” and “Was there anything that exhausted you during the learning activity? If so, what caused the exhaustion?” Furthermore, the interviews addressed specific usability issues observed when the participants completed the application. Each interview lasted approximately 35-40 minutes, and the interview sessions were audio-recorded.

### Analysis

The data were analyzed using different approaches. The average score from the SUS questionnaire was calculated using the procedure described by Brooke [38], presented as mean and SD values, and then given a graded score (A-F) based on the acceptability range. The average adjective score was calculated as recommended [40]. Data on task completion time (efficiency) were gathered from field notes and video recordings and presented with descriptive statistics.

All material (video recordings, field notes from the think-aloud sessions, and transcribed focus group interviews) was analyzed together as recommended by Rubin and Chisnell [25], for completeness and to obtain an overview during analysis. The first author transcribed all audio-recorded material (think-aloud sessions and focus group interviews). The transcribed material was analyzed with the field notes, as recommended by Rubin and Chisnell [25], for completeness and to obtain an overview during analysis. A reflexive thematic analysis [41] was conducted to identify in-depth usability issues, with an emphasis on participants’ experiences. The first author led the analysis of the audio-recorded material and field notes to ensure consistency, but the coauthors reviewed and discussed the analysis until an agreement between the coauthors and the first author was reached.

### Ethical Considerations

Permission was obtained from the head of the nursing study program at the Department of Health and Nursing Sciences at the University of Agder, the Faculty Ethics Committee at the University of Agder, and the Norwegian Center for Research Data (305866).

## Results

### Participants

A total of 9 students responded, and all were included, comprising 7 females and 2 males ranging in ages between 22 and 29 years. All participants previously had taken part in compulsory ISBAR teaching in nursing education. The participants reported their technological competency to be either level 2 (n=5) or level 3 (n=4).

### Overall System Usability Assessment

**Figure 1:**
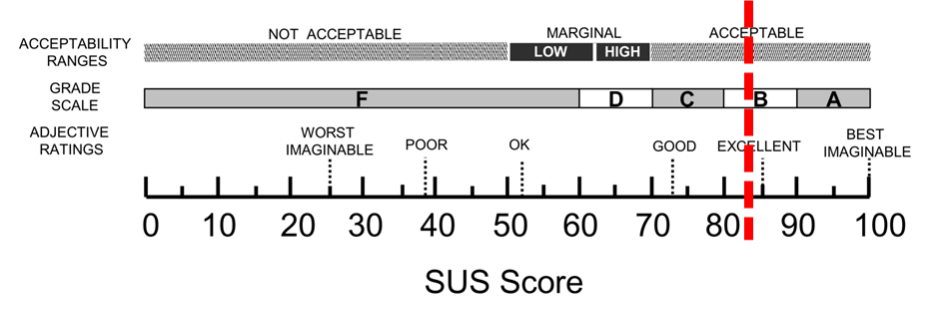
Overall system usability assessment. The vertical dotted red line (83 on the 0-100 scale) shows the mean system usability score (SUS) (n=9) (reproduced with permission from Aaron Bangor [42]). SUS: System Usability Scale.

The overall mean SUS score for the application was 83 (SD 18.8; Figure 1), which rates as *Acceptable* on the acceptability range, *B* on the graded scale, and *Excellent* on the adjective rating scale (Figure 1).

### Time

The 3 groups took 28, 37, and 48 minutes to complete all sequences in the application once, with a mean time of 38 minutes. The group that took 48 minutes had one participant who spent 15 minutes sorting patient information (Table 1, sequence 5), while the other participants used 3 to 5 minutes on this task.

### In-depth Usability Issues (Thematic Analysis)

The qualitative findings were categorized into two themes: (1) more motivational than standard learning activities and (2) technical and comprehension issues.

#### More Motivational Than Standard Learning Activities

All participants recommended this learning activity to others and said it was a motivational way of learning ISBAR. The participants said the application helped them learn ISBAR through the self-instructed exercise, discussions with other participants, observing how others performed the tasks, and when the instructions told them to reflect on their performance together with the others.

It was good to practice ISBAR instead of just reading. It is actually better to do it. It is more like reality and a lot more fun. Communication is a skill. By reading about ISBAR, you will never be good at communicating. It is a skill that must be practiced. By using this, you are practicing communication. You can memorize the letters in ISBAR, but you cannot use it if you haven't practiced.

ID 07, self-reported technological competence level 2

All the participants concluded that the application’s features, such as the automatic visualized feedback, motivated them to complete the exercise. Some of them said that being represented as avatars with their own voices and not having to reveal themselves on camera was a good way to practice. Furthermore, some commented that communication through the virtual phone call made them realize that they needed to speak clearly and loudly.

I feel that I am more invested in it because it is a PC program. It could be a desire for learning or a competitive instinct, but I want to complete this program. I liked the feeling of progression and the structure. Everyone knew what we were going to do, and we knew what to do. It is systematic, and you go on and on and on and on.

ID 02, self-reported technological competence level 3

Some also described it as being closer to clinical practice compared with standard learning activities.

It was simulated in a way that made me feel that I got something out of it, and it was a good way to go from theory to practice. It is like a clinical procedure; you do not know how to do it by reading the procedure, but by doing it. You learn to use ISBAR during clinical practice, but having a good start like this can make you learn faster and better.

ID 01, self-reported technological competence level 3

#### Technical and Comprehension Issues

All groups managed to complete the application and all the users were able to complete the tasks “Familiarization with desktop VR and each other” and “Sort patient information” at the first attempt. Screen transitions were crisp and smooth, with no apparent technical lag times that may have led to negative usability. Through the interviews, most participants said it was easy to follow the application’s flow and complete the tasks.

Technically, it is very easy to understand. For me, it looks like anyone could have managed this. If a technical manager had assisted, that person would not have needed to help them much. It was obvious.

ID 05, self-reported technological competence level 3

However, in 2 of 3 usability tests, the application was restarted. In 1 test, a participant had trouble getting access to the microphone on the computer, so the other participants had to wait until this was solved. During another test, a participant clicked on the “Next” button on the screen at a point when they are supposed to introduce themselves to each other (Table 1, sequence 3). Thus, they were instructed to restart the application. When these 2 issues were resolved, the groups completed the tasks.

The “technical” problems that occurred were frustrating. I was terrified of doing something wrong. I understood that if you clicked “Next,” everyone must start again. So, I got stressed because of the disturbances initially when we had to start over again. Then I thought that I would not ruin it for everyone else. And then I just got even more stressed.

ID 04, Self-reported technological competence level 2

Some participants said they had problems understanding parts of each task. One reason was that they did not hear the voiceover instructions (Table 1, sequences 2, 4, 7, and 9) owing to other participants commenting or asking questions during the instructions. Another reason given was reluctance to open the available pop-up windows to repeat the instructions for fear of appearing slow or incompetent to other participants. Finally, some said that the most prolonged instructions contained too much information (Table 1, sequences 8 and 10), making them forget what was said.

I did not really understand whether we should include everything or not. That was the hardest to understand. I think it was because I did not read the instructions before. I was stressed, feeling the others may read faster than me. And I am slow, so I just had to hurry, right? And then I did not read the instructions.

ID 09, self-reported technological competence level 2

During task completion, 2 of the 9 participants asked for instructions from the observer in the room. The requested instructions were in sequence 5 (Table 1), when it was unclear whether they should answer individually or in a group, and in sequence 11, when someone asked for instructions on how to solve the task regarding whether they should sort all patient information or only some of it.

## Discussion

### Principal Findings

This study aimed to identify the perceived usability of the application as evaluated by second-year nursing students, who found the learning activity to be usable overall, rating it highly, although with some technical and comprehension issues that impeded the experience for some testers.

### Recommended Changes to the Preoperative ISBAR Desktop VR Application

As described, usability issues were found, and it is recommended that such issues be addressed by making changes to the application. Some participants took an unnecessarily long time to complete some tasks, for example, trying to perfect their answers. However, this may be due to the experimental task given and not a usability issue. Nevertheless, it is recommended to impose a time limit for some tasks (Table 1, sequences 5 and 11, with a time limit of 5.5 minutes and 1 minute, respectively) to more accurately reflect the practical context (eg, time pressure, stress, and workload).

Considering that participants were disturbed when other participants talked during the instructions, it is recommended that participants be muted while instructions are given (Table 1, sequences 2, 4, 7, and 9). To avoid the participant clicking on the “Next” button too early, a 10-second delay after the spoken instructions are completed before “Next” can be clicked is recommended. Furthermore, it is recommended that each written instruction sequence and task sequence start with the informative pop-up windows open so that they only can be closed manually to allow all participants to read or reread through the information at their own pace, which is an appealing approach for students [43].

### VR as a Learning Technology

All participants found the VR application to be a motivational way of learning ISBAR. Using desktop VR for learning purposes seems to fit the targeted users, which is perhaps not surprising, as they all were born in the mid-1990s as part of a demographic termed Generation Z [44]. This generation grew up with access to the internet and digital technology from a young age [45]. According to Chicca and Shellenbarger [45], this generation is supported during the learning process when technologically advanced and visually engaging and exciting activities are provided.

Some students said that the application helped them learn the ISBAR approach better than traditional activities, mainly because they could participate actively and experience the training closer to practice. This supports Huang and Liaw [46], suggesting that a well-designed VR learning environment can bridge the gap between theoretical and real-life learning, providing learners with a more authentic learning experience. The results indicate the application’s utility, providing self-reported improvement in the performance of the ISBAR approach compared to conventional training, which could be mediated by the interaction experience and the pedagogical support in the application [47]. Thus, the learning outcome must be further studied using a suitable design to measure the learning effect.

Even if the application’s evaluation primarily was positive, the participants also reported some challenges due to negative stress when task completion did not progress as intended. Technological usability issues affect the participants’ experiences [43,47]. Furthermore, the individual differences in how people react to using VR for learning [46] need to be considered when designing learning activities.

The students stated that they were reluctant to open the pop-up windows for explanations when everyone in the group was watching. Others’ influence has been noted in extant research when participants are observed performing tasks, a phenomenon explained by the social facilitation theory [48,49]. The assumption is that others’ presence can both promote and hinder one’s performance, which also is supported by Strojny et al’s [50] investigation of copresence in VR. In an earlier study, it was suggested that self-paced learning be taken into account during instruction through desktop VR because it generates autonomy [51].

### Methodological Strengths and Limitations

This study’s strength was that the participants were the intended user group, who varied in age, gender, and self-reported technological competence. This variation can enhance the generalizability of the results [25]. Nevertheless, some caution is needed because the participants were self-recruited, which could mean they were overly positive about VR and technology-based teaching [52].

Although the SUS is a recommended tool for evaluating educational technology systems and is suitable for a small sample size [26], the scale was not developed specifically to evaluate learning activities in desktop VR. Therefore, the think-aloud method and focus group interviews were supporting methods in this study.

### Conclusions

The second-year undergraduate nursing students rated the application’s usability as excellent and provided opportunities for active participation, which was motivational and facilitated their perceived learning outcomes. The next version of the application, to be used in a randomized controlled trial and further as a part of clinical preparation in nursing education, will include better technological and comprehension support.

## Appendix

Multimedia Appendix 1 An avatar represents each participant, and the interaction takes place in the desktop virtual reality (VR).

Multimedia Appendix 2 A picture of the screen for the task of sorting patient information. One part of the patient information is shown in the upper left part and is sorted by clicking on one of the ISBAR letters. To the right, the patient information already sorted is displayed.

## Abbreviations

ISBAR: introduction, situation, background, assessment, and recommendation
SUS: System Usability Scale
VR: virtual reality
4C/ID: four-component instructional design
