# Supplementary figures and images for: Usability Evaluation of the Preoperative ISBAR (Identification, Situation, Background, Assessment, and Recommendation) Desktop Virtual Reality Application: Qualitative Observational Study
Source: JMIR Hum Factors. 2022 Dec 29;9(4):e40400. doi: 10.2196/40400 (PMC9837706; doi:10.2196/40400)

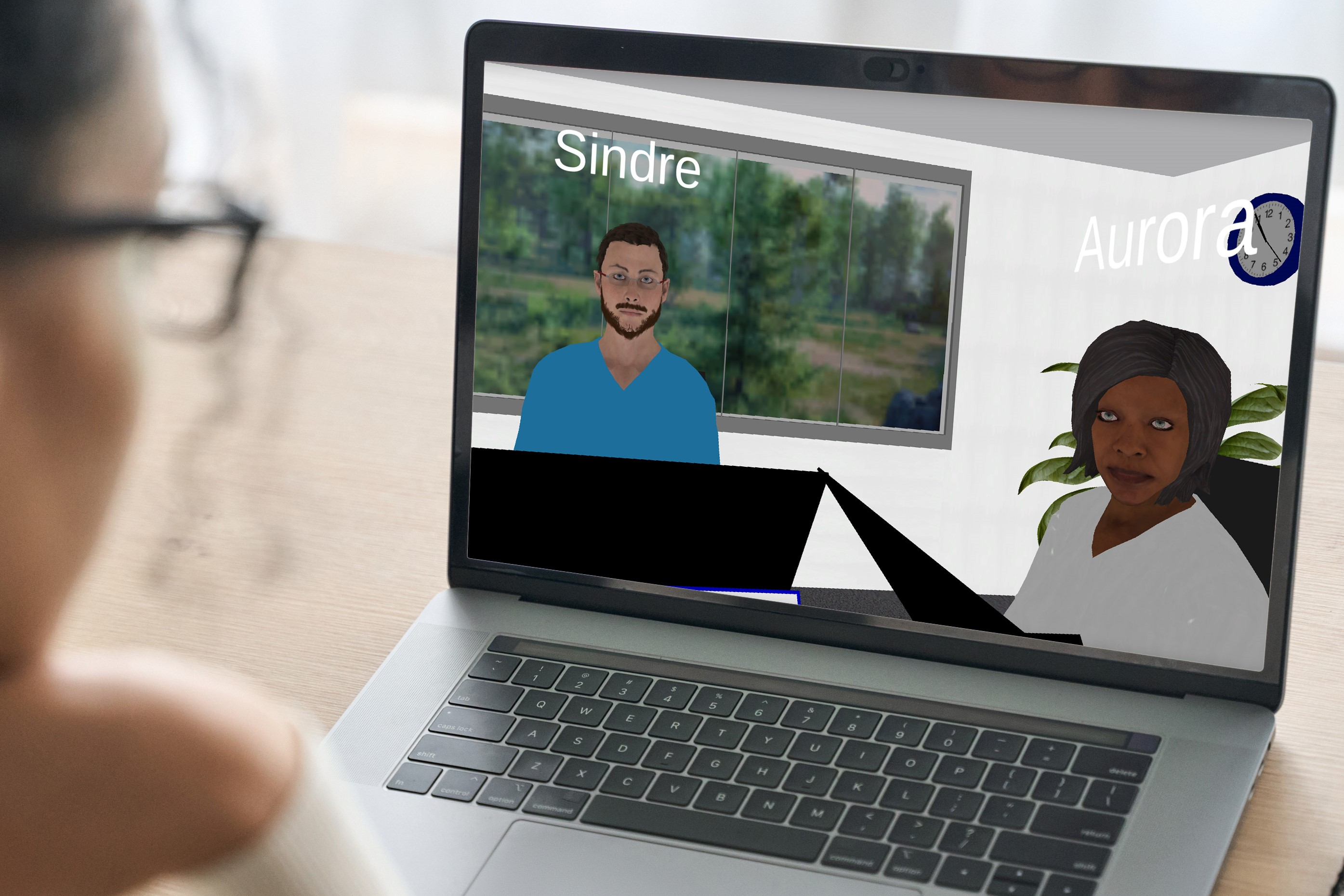

Supplement: Multimedia Appendix 1 [file humanfactors_v9i4e40400_app1.png]

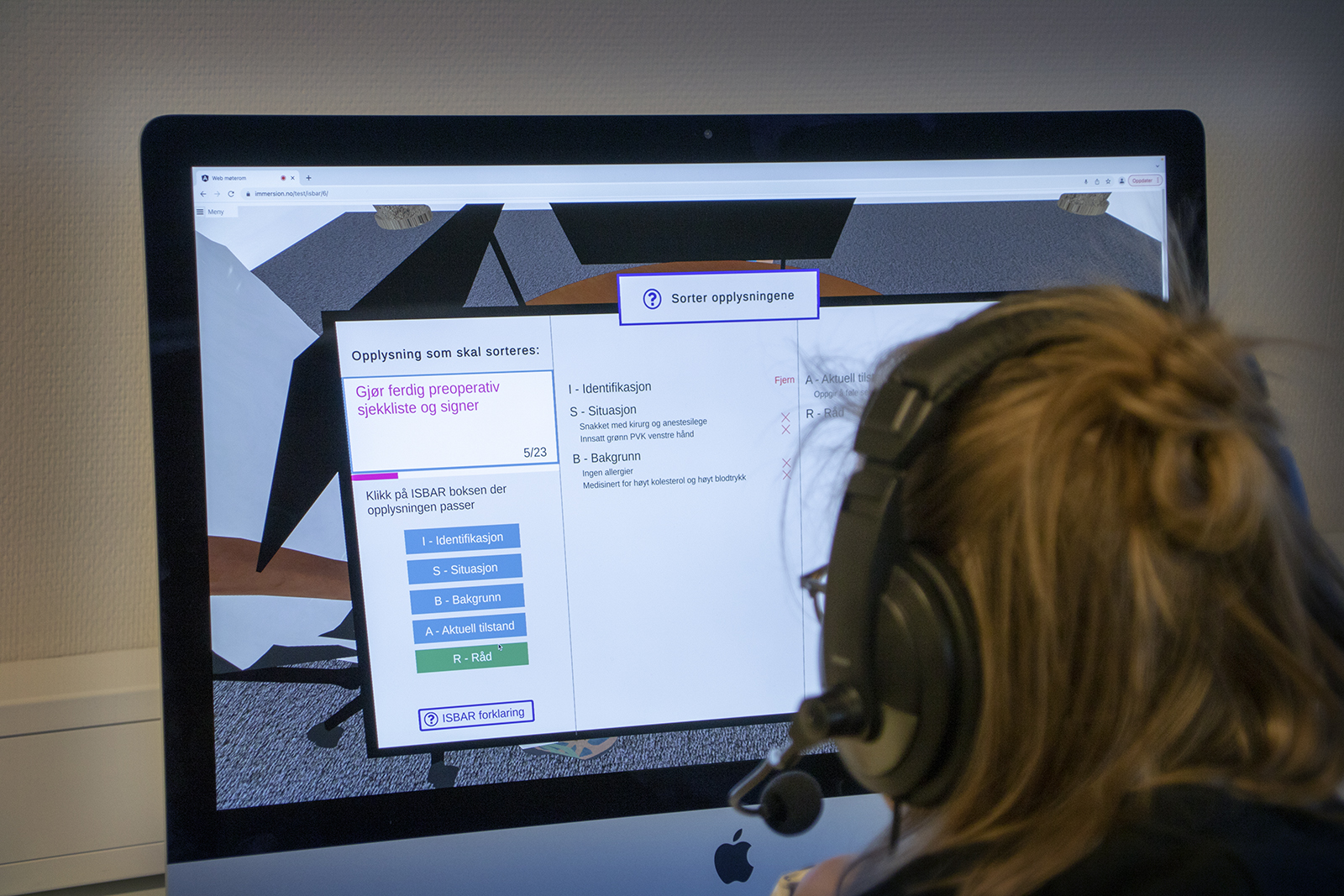

Supplement: Multimedia Appendix 2 [file humanfactors_v9i4e40400_app2.png]
